# VMP1 Establishes ER-Microdomains that Regulate Membrane Contact Sites and Autophagy

**DOI:** 10.1371/journal.pone.0166499

**Published:** 2016-11-18

**Authors:** Luis-Carlos Tábara, Ricardo Escalante

**Affiliations:** Instituto de Investigaciones Biomédicas Alberto Sols; C.S.I.C./U.A.M.; 28029-Madrid, Spain; Institut Curie, FRANCE

## Abstract

The endoplasmic reticulum (ER) regulates organelle dynamics through the formation of membrane contact sites (MCS). Here we describe that VMP1, a multispanning ER-resident protein involved in autophagy, is enriched in ER micro-domains that are in close proximity to diverse organelles in HeLa and Cos-7 cells. These VMP1 puncta are highly dynamic, moving in concert with lipid droplets, mitochondria and endosomes. Some of these micro-domains are associated with ER sliding events and also with fission events of mitochondria and endosomes. VMP1-depleted cells display increased ER-mitochondria MCS and altered mitochondria morphology demonstrating a role in the regulation of MCS. Additional defects in ER structure and lipid droplets size and distribution are consistent with a more general function of VMP1 in membrane remodeling and organelle function. We hypothesize that in autophagy VMP1 is required for the correct morphogenesis of the omegasome by regulating MCS at the site of autophagosome formation.

## Introduction

The endoplasmic reticulum (ER) is in close proximity with most organelles, establishing membrane contact sites (MCS) that facilitate signaling events and trafficking of lipids and ions [1–3]. The role of MCS in autophagy is now emerging. The ER forms a specialized structure, the omegasome that cradles the phagophore (also known as isolation membrane) as it elongates to form a mature double membrane autophagosome. Electron tomography studies have revealed MCS between ER and the phagophore membrane [4, 5]. The omegasomes are enriched in the signaling lipid PtdIns3-P, generated by the class III PtIns3-kinase VPS34, and this signaling event triggers the recruitment of autophagic proteins [6, 7]. The membrane source that elongates the phagophore may come from the ER itself and from vesicles originating from ERGIC [8], Golgi and recycling endosomes [9, 10], some of them containing Atg9 and lipidated LC3. In addition, it has been proposed that autophagosomes are formed at ER-mitochondria contact sites [11]. Autophagosome formation also requires the close proximity of other organelles or ER regions such as lipid droplets (LDs) [12, 13], late endosomes [14] and ER-exit sites (ERES) [15, 16]. Recent ultrastructural electron tomography studies have shown the presence of MCS between the phagophore and late endosomes, Golgi complex and mitochondria [17].

VMP1 (Vacuole membrane protein 1) is a conserved multispanning transmembrane protein localized to the ER in *Dictyostelium* [18], *Drosophila* [19], *Arabidopsis* [20] and HeLa cells [21]. The intracellular localization pattern of VMP1 fused to GFP is complex in HeLa cells showing scattered puncta over the ER-tubules. Although autophagy flux is blocked in VMP1-deficient cells, the PtdIns3-P production and recruitment of the autophagy machinery can still occur but the completion of the autophagosome is impaired [22]. Indeed, levels of PtdIns3-P are abnormally high in the absence of VMP1, and accumulation of large omegasomes and LC3 puncta is observed in mammalian cells [21–23], *Caenorhabditis* [23] and *Dictyostelium* [24, 25]. These results support the model that VMP1 is required for the correct structure of the omegasome and/or for the adequate capacity of the phagophore to elongate and become a functional autophagosome. The inactivation of VMP1 in model organisms causes pleiotropic phenotypes, leading to the hypothesis that VMP1 may play additional non-autophagic roles. Studies in *Dictyostelium* [18], plants [20] and *Chlamydomonas* [26] suggest that VMP1 is involved in processes as diverse as protein secretion, endo- and phagocytosis, osmoregulation, cytokinesis, regulation of organelle's function and morphology. How a single protein can regulate such a wide range of processes is not known. We now report that VMP1 may be a common element in different ER-organelle contact sites and regulates the size of the ER-mitochondria contacts, which may affect diverse cellular processes. We also hypothesize that VMP1 might orchestrate the multiple interactions among the omegasome, the autophagic machinery and the organelles required for phagophore elongation.

## Materials and Methods

### Plasmids

VMP1 and ΔNt-VMP1-encoding DNA sequences were amplified by PCR from HeLa cDNA using the following primers and cloned into the vector pEGFP-N1 using the restriction enzymes XhoI/EcoRI and EcoRI/SalI respectively. VMP1 Fw: 5'- GCG CTC GAG ATG GCA GAG AAT GGA AAA AAT TGT-3'; Rv: 5'-GCG GAA TTC CTT TAG TTT TCT CCT CTG AGT T-3'). ΔNt-VMP1: Fw: 5'-GTG GAA TTC GAA CCA TGA TCC TTG TAA TCT TGA AGG AAT GG-3'; Rv: 5'-GCG GGT CGA CCG TTT AGT TTT CTC CTC TGA GTT C-3'.

Other plasmids were purchased from Addgene: Sec61-mCherry (Addgene #49155); Rab5-mCherry (Addgene #49201); Rab7-mCherry (Addgene #61804), MitoBlue, (Addgene #49151); DFCP1-Flag (Addgene #45917); DFCP1-GFP (Addgene #38629).

### Cell growth, transfections and treatments

HeLa and Cos-7 cells were gifts from Dr. Alberto Muñoz and Dr. Juan Bernal from Biomedical Research Institute, Madrid, Spain. They were grown in DMEM (Dulbecco's Modified Eagle Medium) with 10% FBS and 5% of Penicilin/Streptomycin in 5% carbon dioxide at 37°C. For experiments, cells were seeded in supplemented DMEM 24 hours prior transient transfection in 24 well plate (Corning) or μ-Slide 8 Well (IBIDI). Cells were at 80% of confluence at the moment of the transfection. Lipofectamine 2000 (Invitrogen) and DNA were diluted in Opti-MEM serum free medium (Life techonologies) following the manufacturer's instructions. After 6 hours, Opti-MEM free medium was replaced for supplemented DMEM. Transfected cells were observed 24 hours after transfection.

Knockdown was performed by reverse transfection of VMP1 siRNA (Ambion, S37754 and S37756), ATG5 (Ambion s18158) or control siRNA (Ambion, control#2) with Lipofectamine RNAiMAX (Invitrogen, 13778–150), according to the manufacturer's instructions. Cells were silenced again with the same siRNAs after 48 h from the first transfection and the assays were carried out 48h after the second transfection.

For nocodazole (NC) treatment, Cos-7 and HeLa cells were incubated with NC (Sigma-Aldrich M1404)-containing DMEM (2,5 μg/ml) for 3 hours. For Oleic acid (OA) treatment, Cos-7 and HeLa cells were incubated with OA (Sigma-Aldrich O1008)-containing DMEM (500μM) for 16 hours. Treatment with cell dyes: living cells were treated with MitoTracker Red CMXRos (Molecular Probes) for 20 minutes at 100 nM in DMEM at 37°C. Living cells were treated with BODIPY 558/568-C12 (Molecular Probes) with 1 μg/ml (in DMEM) for 45 minutes. Cells were washed with DMEM prior observation. For induction of autophagy cells were incubated in EBSS medium for two hours prior fixation.

### Western-Blot

Cells were homogenized in RIPA buffer, then separated by SDS-PAGE under reducing conditions and transferred to nitrocellulose membranes (GE Healthcare), blocked with 5% skimmed milk in TBS/0.5% v/v Tween 20 for 1 h, and washed with TBS/Tween 20. Immunoblots were performed using the following primary antibodies: anti-VMP1 (Cell Signaling #12929), anti-ATG5 (Cell Signaling #2630), anti-TIM23 (BD Biosciences #611222) and anti-GAPDH (Enzo ADI-CSA-335). Horseradish peroxidase-conjugated secondary antibodies were purchased from Santa Cruz Biotechnology (goat anti-mouse and goat anti-rabbit). Blots were developed with the enhanced chemiluminescence method following the manufacturer's instructions (Amersham). For western-blot analysis of LC3 (Cell Signaling #2775S) total cell lysates were isolated using RIPA buffer from cells incubated in complete (DMEM) or starvation medium (EBSS) for 4 hours in the presence or absence of 50 μM chloroquine.

### Confocal and transmission electron microscopy (TEM)

Confocal images were acquired using an inverted Zeiss LSM 710 laser-scanning microscope. For confocal visualization, cells were incubated in complete medium (DMEM, Dulbecco's modified Eagle's medium) or starvation medium (EBSS, Earle's balanced salt solution) for 2 h, fixed with 4% paraformaldehyde, blocked, immunostained and mounted with ProLongGold (Invitrogen, P36934) for visualization. Primary antibodies used were: Sec31 (BD Biosciences #612350), Ergic53 (SantaCruz #sc-365158), GM130 (BD Biosciences #610822), TGN46 (Merck #ABT95), Catalase (Calbiochem #219010), DYKDDDDK-Flag (Cell Signaling #2368) and LC3 (Cell Signaling #2775S).

For *in vivo* imaging, cells expressing VMP1-GFP and specific organelle markers were imaged in a single plane of focus for 3 min with images taken every 1 second.

For conventional TEM, control and silenced cells were grown in DMEM on 60 mm plates. Cells were fixed with 4% PFA and 2% glutaraldehyde (GLA) in 0.1 M phosphate buffer (PB, pH 7.4) for 90 min at RT. Post-fixation was carried out with 1% OsO_4_ and 1.5% K^3^Fe(CN)^6^ in water at 4°C for 1 h. Samples were dehydrated with acetone and in situ flat-embedded in Epoxy, TAAB 812 Resin (TAAB Laboratories) according to standard procedures. After polymerization, resin sheets containing the cell monolayers were detached from the substrate and mounted onto resin blocks to obtain orthogonal or parallel (from the bottom to the top of the cell) 80-nm ultrathin sections. The sections were deposited onto slot grids, stained with saturated uranyl acetate and lead citrate and examined at 80 kV in a Jeol JEM-1010 electron microscope. Images were recorded with a TemCam-F416 (4Kx4K) digital camera from TVIPS.

For immunoelectron microscopy, transfected cells with VMP1-GFP were fixed with 2% PFA and 0.2% GLA or 4% PFA and 0.05% GLA in 0.1 M PB for 2 h at RT and kept in 1% (w/v) PFA in PB at 4°C. Subsequently, cells were embedded in 10% (w/v) gelatin and cryoprotected overnight in sucrose 2.3 M. Specimens were rapidly frozen in liquid nitrogen and cryosectioned with a Leica EM FCS cryo-ultramicrotome at -120°C. For immunogold labeling, thawed 90-nm thick cryosections were incubated with 20 mM glycine for 5 min at RT to quench free aldehyde groups and with 10% FBS for 5 min at RT to block nonspecific binding. Then, GFP antibody were incubated for 30 min at RT followed by protein A conjugated to 15-nm gold particles (EM Laboratory, Utrecht University, The Netherlands) or goat anti-mouse IgG conjugated to 15-nm gold particles (Aurion) for 30 min at RT. The primary antibodies and gold conjugates were diluted in PBS containing 5% FBS.

### Quantification and statistical analysis

For the colocalization analysis typically at least 20–30 cells were analyzed manually from 3 independent transfections (approximately 10 cells per transfection). Morphometric quantifications of TEM images from two independent experiments were performed manually with the help of imageJ64 to determine the mitochondrial perimeter and contact distances. LD clustering was calculated manually according to [27]. A Lipid droplet cluster was defined as groups of 3 or more lipid droplets in a circular area of 2 μm in diameter. In all cases the mean and the standard deviation (SD) were obtained from the averages of the independent experiments. Significance was analyzed using the Student *t* test.

## Results

### VMP1 micro-domains are associated with ER sliding events

**Fig 1:**
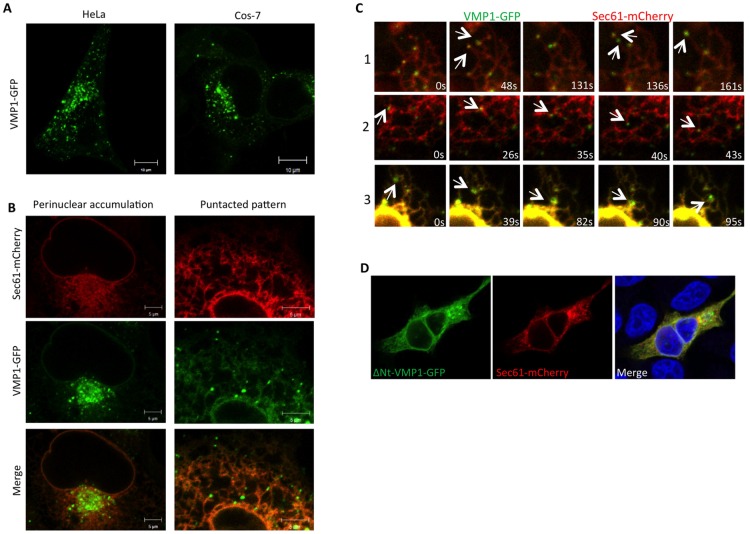
VMP1 is associated to ER sliding events. (A) HeLa and Cos-7 cells transfected with a plasmid encoding VMP1 fused to GFP (VMP1-GFP) and visualized by confocal microscopy show a similar pattern. (B) Analysis of confocal microscopy of Cos-7 cells expressing VMP1-GFP and the ER marker Sec61. The punctated pattern is evident in the peripheral tubules and the perinuclear area. (C) *In vivo* time-lapse confocal microscopy of cells expressing VMP1-GFP and Sec61-mCherry. Different examples of sliding events are shown in frames. The corresponding movies are available as supplementary material (S1–S3 Movies). (D) ΔNt-VMP1-GFP were transfected in HeLa cells and colocalized with the ER marker Sec61 by confocal microscopy. The protein distributes throughout the ER loosing the punctated pattern in HeLa cells.

We used a C-terminal GFP fusion to human VMP1 that has been shown to be fully functional in *Dictyostelium* and *Arabidopsis* [18, 20] to analyze VMP1 dynamics in HeLa as well as in Cos-7 cells, which allow a more precise analysis of the ER structure. We first confirmed that VMP1-GFP display the same localization in both cell types consisting of a distinct punctated pattern over discrete regions of the tubular ER and an enrichment of the marker at the perinuclear area (Fig 1A). Increasing the intensity of the color channel allowed the visualization of low levels of VMP1-GFP along the whole ER network (Fig 1B). Using time-lapse confocal microscopy of living cells we found that VMP1 puncta are highly dynamic and frequently associated with ER movements known as sliding events [28–30]. Several examples are displayed in Fig 1C and S1–S3 Movies, in which VMP1 puncta often locate either at the tip of tubules as they move or at sites where the tubules are pulled or stretched.

The topology of VMP1 has been reported for the Arabidopsis thaliana homologues KMS1 and KMS2 [20]. The high conservation of the amino-acid sequence suggests that the mammalian VMP1 likely shares the same topology with six transmembrane regions. Both the N-terminus and the C-terminus are predicted to face the cytoplasmic side. While the C-terminal region contains the putative ER-retention signal (XKXX signature), the N-terminal one includes a conserved sequence with the potential to form an amphipathic alpha helix (predicted by *in silico* analysis). Since amphipathic helices affect membrane curvature [31], we tested the functionality of this region of VMP1 by deleting the first 61 amino-acids corresponding to the predicted N-terminal cytoplasmic tail. Interestingly, the punctuated pattern and the perinuclear accumulation are lost with the truncated form (ΔNt-Vmp1-GFP), which is distributed uniformly throughout the whole ER in HeLa cells (Fig 1D). These results suggest that the N-terminal region of the protein is required for the formation of VMP1 puncta and confirm the ER localization of VMP1. Further studies will be required to address the mechanism by which this region mediates the formation of these VMP1 puncta.

**Fig 2:**
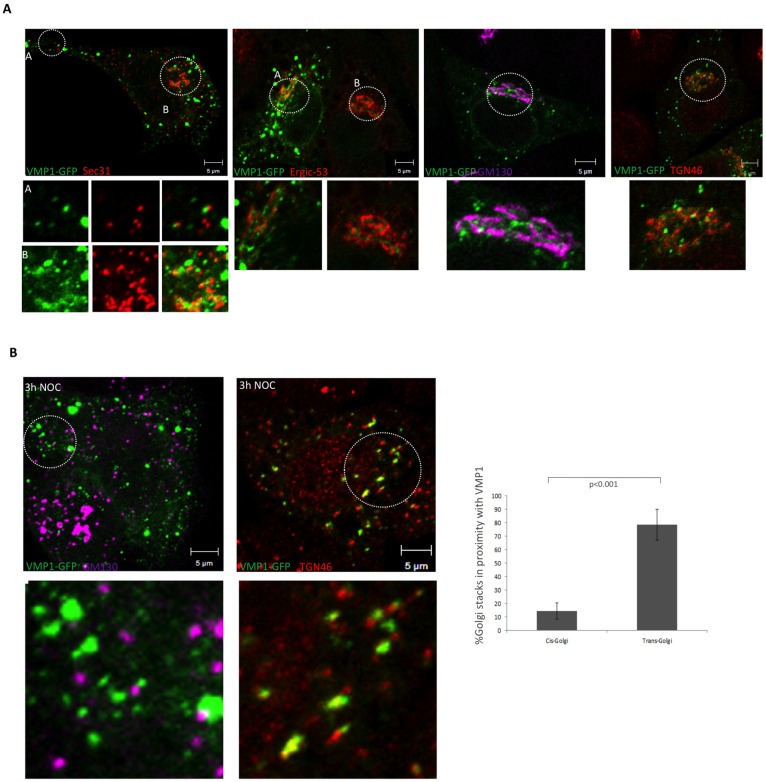
Analysis of colocalizations at the perinuclear area. (A) Confocal microscopy analysis of HeLa cells expressing VMP1-GFP and incubated with antibodies that recognize ERES (Sec31), ERGIC (Ergic53) and Golgi (GM130 and TGN46). No colocalizations were observed, although some regions showed close proximity. (B) Cells expressing VMP1-GFP were treated with nocodazole prior to fixation and detection of Golgi markers by confocal microscopy. Close localization and some degree of colocalization were observed with the trans-Golgi marker TGN46. The mean and the SD were obtained from two independent experiments (approximately 5–7 cells per experiment).

The VMP1-GFP signal at the perinuclear area consists of more static puncta over a diffuse signal. We used markers of ERES (ER-exit sites), ERGIC (ER-Golgi intermediate compartment) and Golgi for colocalization analysis. VMP1 was closely associated but clearly distinct from ERES, ERGIC and Golgi (Fig 2A). Treatment of cells with the microtubule polymerization inhibitor nocodazole disrupts Golgi structure leading to a dispersal of the cisterna, which facilitates colocalization studies. We treated HeLa cells expressing VMP1-GFP with nocodazole and used cis- and trans-Golgi markers for confocal analysis. While the cis-Golgi marker shows minor colocalization with VMP1, the trans-Golgi one presented a more closely association after nocodazole treatment (Fig 2B).

### VMP1 puncta dynamically associates with organelles

**Fig 3:**
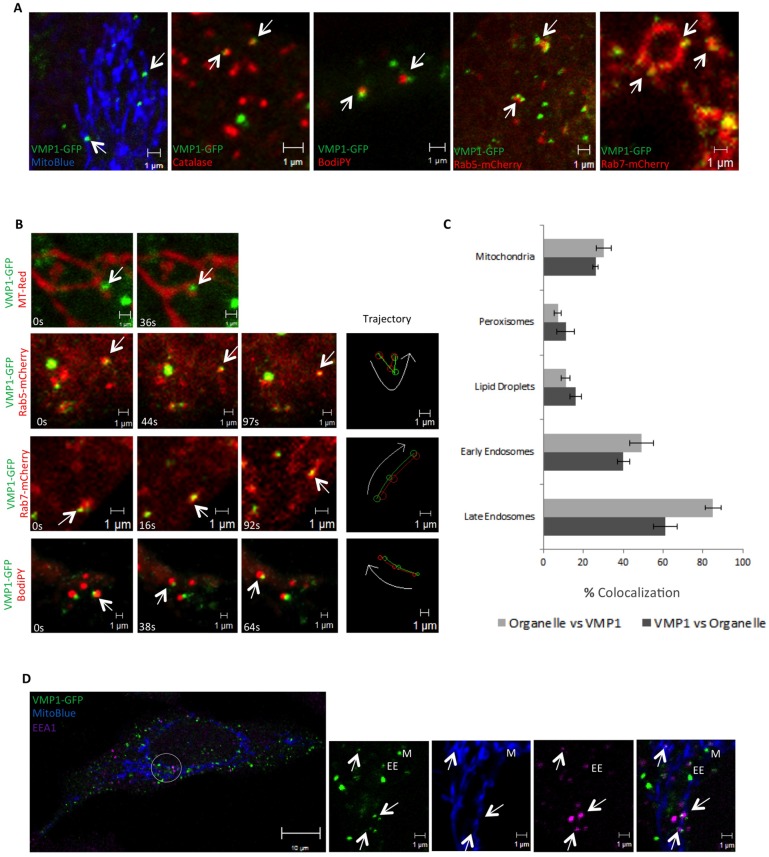
VMP1 establishes close contacts with organelles. (A) Confocal analysis of colocalization of VMP1-GFP expressing HeLa cells with different organelle markers as specified in the images. Similar results were obtained in Cos-7 cells (not shown). (B) *In vivo* time-lapse microscopy showing examples of concerted movement between VMP1 and mitochondria, lipid droplets and endosomes (corresponding to S4–S7 Movies). (C) Quantification of the proportion of VMP1 puncta in contact with organelles (and the proportion of organelles in contact with VMP1). The mean and standard deviation (SD) were obtained from three independent transfections (approximately10 cells per transfection were analyzed). (D) Triple colocalization of VMP1 puncta with mitochondria and endosomes. VMP1 puncta colocalizing or in close proximity to mitochondria and endosomes are marked by M or EE respectively. Triple colocalizations are marked by arrows.

ER sliding dynamics can result from ER remodeling events as well as from the pulling effect of a trafficking organelle that is connected to the ER by MCS [32]. Therefore, we investigated if VMP1 puncta could be associated with other organelles and if this association was related with membrane remodeling events. We analyzed by confocal microscopy the spatial localization of VMP1 together with several organelle markers. Remarkably we found that VMP1 puncta were closely associated with mitochondria, peroxisomes, Rab5 and Rab7-marked endosomes and lipid droplets (Fig 3A). It should be noted that not all VMP1 puncta appeared in contact with these organelles, and reversely, not all organelles were in contact with VMP1. Quantification of these close-contacts indicate that the frequency of association with VMP1 puncta differs for each organelle (Fig 3C), with Rab7-containing late endosomes showing the most frequent colocalization with VMP1 (59% of VMP1 puncta colocalize with Rab7 and 85% of Rab7 endosomes colocalize with VMP1). Triple marker staining showed that one VMP1 puncta can be associated with more than one organelle (Fig 3D). For example, VMP1 puncta can be found in closed association with mitochondria and endosomes and some of the puncta simultaneously colocalize with both organelles.

**Fig 4:**
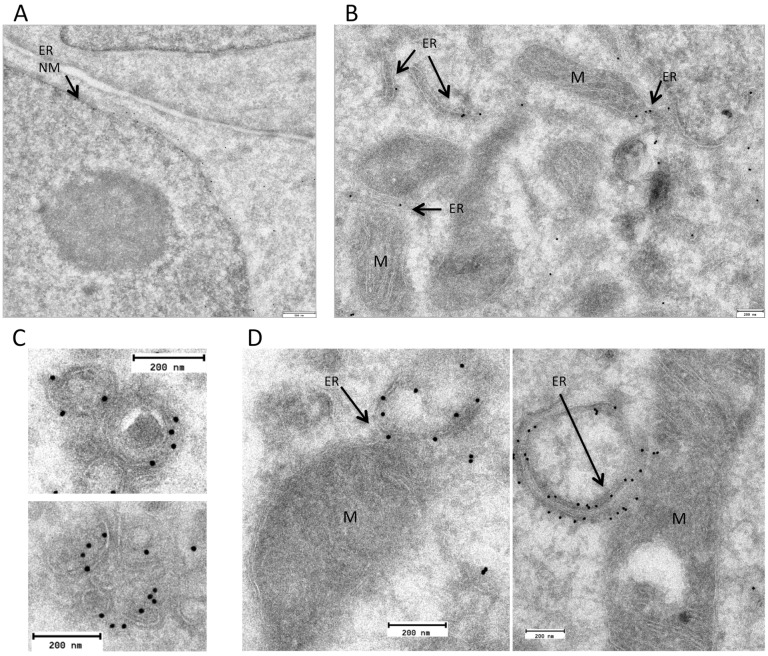
VMP1 is an ER protein in close proximity to vesicles and mitochondria. Cells expressing VMP1-GFP were subjected to inmuno-gold labeling with an antibody against GFP and visualized by TEM. Labeling is found in nuclear membrane (NM) (A) and ER tubules (B) as well as in close proximity to vesicles (C) and mitochondria (M)

Time-lapse confocal microscopy of living cells was next performed to study the dynamics of the association of VMP1 puncta with these organelles. Remarkably, we observed that VMP1-organelle contacts remain closely connected as VMP1 puncta moved in concert with mitochondria, lipid droplets and endosomes (Fig 3B, S4–S7 Movies), thus strongly suggesting that these VMP1-enriched ER puncta are tethered to different organelles. Besides their coordinated movement, we also found that VMP1-organelle contacts are dynamically associated with organelle remodeling processes such as fission events. These events are rare but several examples of mitochondria and endosome fissions were found (S8–S10 Movies). Following the fission event, VMP1 puncta often seemed to split remaining at both edges of the fragmented organelles. The same behavior was observed during the concerted movement of VMP1 with lipid droplets (S11 Movie). These phenomenological observations do not imply that VMP1 is directly involved in the fission machinery, an issue that will require further investigation. Taken together, these results show that VMP1 puncta associate dynamically with diverse organelles as they move and remodel. To further confirm the close association of VMP1 with organelles Immunogold TEM were also performed in cells expressing VMP1-GFP. We found nuclear membrane and ER tubules decorated with gold particles (Fig 4A and 4B) and also in close proximity to vesicle-like structures (Fig 4C) and mitochondria (Fig 4D). The distance between some of these ER-labeled features and organelles are within 30 nm, which is compatible with possible MCS [33]. Nevertheless, since we have not characterized these regions biochemically we can not ascertain at this point that VMP1 is part of any of the described tethering complexes that mediate MCS [1].

### A small proportion of VMP1 puncta are engaged in autophagy

**Fig 5:**
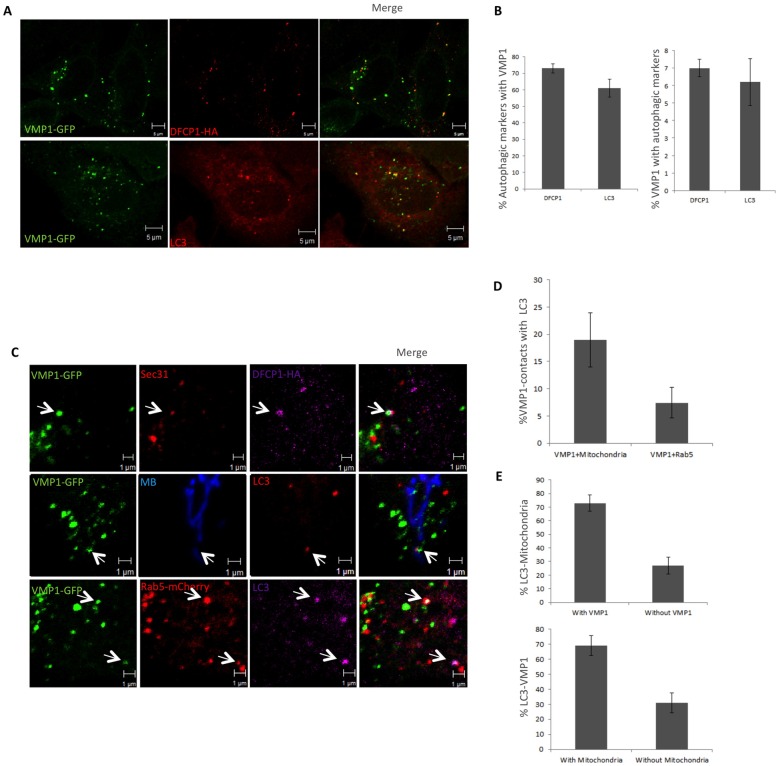
VMP1 associates simultaneously with organelles and autophagosomes. (A) Confocal microscopy analyses showing the level of colocalization of VMP1 puncta with the omegasome marker DFCP1(detected by expression of DFCP1-HA and subsequent inmunolocalization using anti-HA antibody) and LC3 (detected with LC3 Ab). Cells were under starvation conditions to induce autophagy (EBSS during 2 hours) (B) Quantification of VMP1 colocalizations with DFCP1 and LC3. The mean and standard deviation (SD) were obtained from three independent transfections (approximately 7 cells per transfecction). (C) Confocal analysis of colocalization between VMP1-GFP, autophagosome markers (LC3 or DFCP1) and organelles (Mitochondria labeled by MitoBlue, ERES detected by Sec31 Ab and early endosomes labeled by Rab5-mCherry). (D, E) Quantification of colocalization between VMP1 and mitochondria or endosomes as indicated in the figure. The mean and SD were obtained from 2 independent transfections (approximately 10 cells per transfection).

Since VMP1 has been previously associated with autophagosomes we have now extended these studies by analyzing the occurrence of VMP1-autophagosome colocalizations in the context of organelle contacts. Our results first indicate that autophagic markers DFCP1 and LC3 localize extensively with VMP1 under starvation conditions (Fig 5A) as expected from previous studies that showed colocalization of VMP1 with ULK1 [34]. Quantification analysis (Fig 5B) showed that most autophagic structures colocalize with VMP1 puncta. However only around 5% of VMP1 puncta colocalized with the autophagic markers suggesting that most VMP1 puncta are not engaged in autophagy, which is in agreement with our previous observations showing close association of VMP1 with many different organelles. Interestingly, triple colocalization analysis showed that DFCP1 or LC3 autophagic markers can be found simultaneously in close proximity with VMP1, mitochondria, endosomes and ER-exit sites (ERES) (Fig 5C). When considering only the pool of VMP1-mitochondrial colocalizations, we observed that 20% are positive for LC3 while only around 7% of VMP1-Rab5 positive puncta are in close proximity with LC3 (Fig 5D). The increase in the colocalization proportion of LC3 with VMP1-mitochondria (20%), with respect to the entire VMP1 puncta (5%), is in agreement with the proposed function of ER-mitochondria contact sites as prominent sites of autophagosome formation. Nevertheless, although small, a proportion of VMP1-Rab5 structures colocalized with LC3, suggesting that autophagosomes might also arise from these contacts.

In addition, when considering only the LC3-mitochondria contacts we found that a large proportion of these colocalizations also contain VMP1 (74%) (Fig 5E). Consistently, around 69% of LC3-VMP1 puncta colocalize with mitochondria (Fig 5E). All together these studies indicate that autophagy-related VMP1 puncta associates frequently with ER-mitochondria contacts but these associations only account for a low proportion of all VMP1 puncta.

### VMP1 depletion block autophagy and affects ER structure and lipid droplets

**Fig 6:**
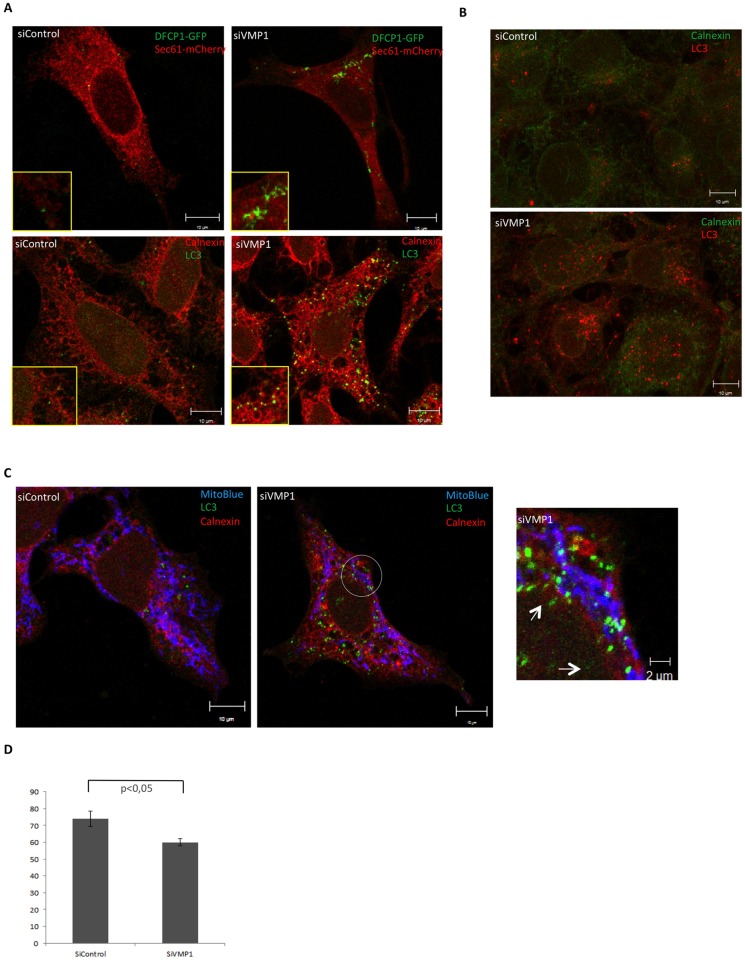
VMP1 depletion block starvation-induced autophagy in HeLa and Cos7 cells. (A) Confocal microscopy analysis of control and VMP1-depleted HeLa cells expressing the omegasome marker DFCP1 and the ER marker Sec61. Aberrant morphology of the omegasome marker DFCP1 is shown. Detection of LC3 and the ER-marker Calnexin in control and VMP1-depleted HeLa cells to show the accumulation of LC3 structures at the ER. (B) Detection by confocal microscopy of LC3 and the ER-marker Calnexin of control and VMP1-depleted Cos-7 cells to show the accumulation of LC3 structures at the ER. (C) Triple colocalization of Mitochondria, ER and LC3 in control and VMP1-depleted HeLa cells. Below the quantification of colocalization events between LC3 and mitochondria is shown. All experiments were under starvation conditions to induce autophagy (EBSS during 2 hours). The mean and the SD were obtained from two independent transfections (approximately 10 cells per transfection) and analyzed using the Student *t*-test.

The association of VMP1 with ER, organelles and remodeling events, and the large proportion of VMP1 puncta not associated with autophagy suggest that this protein could play additional roles in ER structure and organelle's function. To address this hypothesis the levels of VMP1 were down-regulated by siRNA-mediated RNA interference (RNAi) in HeLa and Cos-7 cells. We used two different hairpins and first checked the level of inhibition (S1A Fig) and the impact on autophagy (S1B Fig), the only phenotype described so far for VMP1 depletion in mammalian cells [21]. As previously described for HeLa cells [21] we found a blockade in autophagy in both cell types at starvation conditions as determined by the LC3 turnover assay (S1B Fig). This blockade is revealed by the presence of high levels of LC3II in the presence and absence of lysosome inhibitors. Interestingly Cos-7 cells showed a blockade only under starvation conditions suggesting that VMP1 might not be required for basal autophagy in Cos-7 cells. Confocal analyses of autophagic markers confirmed the abnormal morphology of the omegasome marker DFCP1 in VMP1-depleted HeLa cells [21] (Fig 6A). We also found an extensive colocalization of the ER with accumulated LC3 in both cell types (although more clearly shown in HeLa cells) as if autophagic structures were trapped at the ER (Fig 6A and 6B), most likely as a consequence of altered MCS (see below). The accumulated LC3 structures are often present in colocalization or close proximity to mitochondria (Fig 6C), suggesting that they remain at ER-mitochondria contact sites, from which autophagosomes have been reported to arise [11]. Quantification analysis (Fig 6D) showed only a slight decrease in the level of colocalization between LC3 and mitochondria in VMP1-depleted cells indicating that VMP1, although present in close proximity as described above, may not be required for establishing the ER-mitochondria contacts (see below for further explanation).

**Fig 7:**
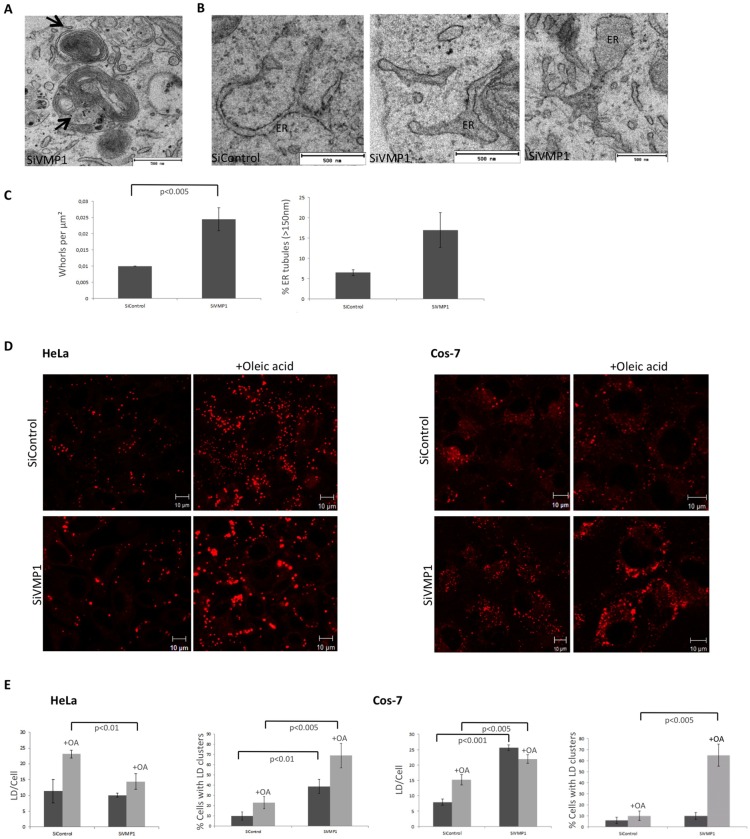
VMP1 dependent alterations in ER structure and lipid droplets. (A) TEM image of whorl structures from VMP1-depleted Cos-7 cells. (B) TEM image of altered ER structures in VMP1-depleted cells. (C) To analyze the abnormal ER features the mean and the SD were obtained fromof two independent inhibitions using 50 sections of 25 μm^2^ for each condition (25 sections per inhibition). (D) Control and VMP1-depleted HeLa and Cos-7 cells were treated with oleic acid and stained for LDs with Bodipy. Representative images of at least three independent experiments are shown. (E) Quantification analysis of LD number and clusters. The mean and the SD were obtained from three independent experiments (approximately 10 cells per experiment). The level of significance were analyzed using the Student *t*-test.

Once confirmed the defect in autophagy, we next checked for additional defects upon VMP1depletion. Analysis of the ER-structure from VMP1-depleted cells by using confocal microscopy and fluorescent ER-markers did not reveal any gross morphological change (not shown). However, TEM analyses of VMP1-depleted Cos-7 cells showed the frequent presence of whorl structures (Fig 7A and 7C), similar to the ones described in *Dictyostelium* autophagy-deficient cells [18, 35] and also what appears as dilated ER (Fig 7B and 7C). Taken together these results suggest that VMP1 plays a role in ER structure as suggested previously in the study of a *Dictyostelium discoideum* Vmp1 mutant that showed fragmented ER [18]. It has been reported that altered ER-LDs MCS lead to changes in LD morphology in *S*. *cerevisiae* [36]. We therefore analyzed the pattern of LDs in VMP1-depleted cells and found changes in the size and distribution of LDs (Fig 7D and 7E), although the phenotype differs between HeLa and Cos-7 cells. In HeLa cells the lack of VMP1 leads to the accumulation of clusters of LD with or without oleic acid and the LD appeared larger in size. However in Cos-7 cells both the number of LD and the distribution in clusters were altered.

### Vmp1-depleted cells have defects in mitochondria morphology and ER-mitochondria contact sites

**Fig 8:**
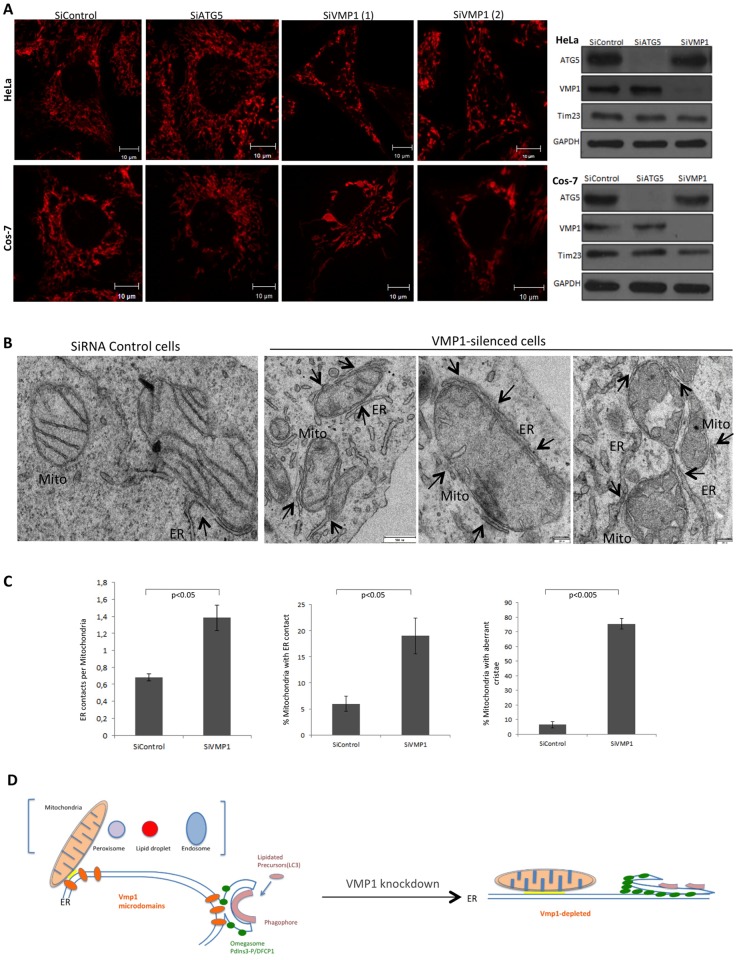
VMP1 regulates ER-mitochondria MCS. (A) Hela and Cos-7 cells were transfected with siRNAs of control, Atg5 and VMP1 (two different hairpins), stained with mitotracker and analyzed by confocal microscopy. Alterations in size and morphology of mitochondria were observed in VMP1-depleted cells. Representative images of at least three independent experiments. To the right, a representative western-blot analysis of the mitochondrial protein Tim23 form HeLa and Cos-7 cells treated with the different siRNAs is shown. Representative western blots of three independent experiments are shown. (B) TEM analysis of control and VMP1-depleted Cos-7 cells show altered mitochondria with dilated cristae and abundant MCS (marked by arrows). (C) Several parameters of MCS and mitochondria morphology between control and VMP1-depleted cells are compared. The mean and the SD were obtained from two independent inhibitions (150 and 100 mitochondrial profiles respectively were analyzed). (D) Model of VMP1 function regulating MCS of multiple organelles and the impact in autophagy. Accumulation of PtdIns3P and autophagic structures at the ER as a consequence of enlarged VMP1 depletion.

The ER-mitochondria contact sites are essential for Ca2+ and lipid traffic and have been proposed to be the platform from which the autophagosome emerges [11]. Therefore, we aimed to determine whether the lack of VMP1 affects the morphology, size or distribution of mitochondria. HeLa and Cos-7 cells were transfected with control and VMP1 siRNAs. Inhibition of Atg5 was also included in the analysis to determine if the defects were VMP1-specific or could be attributed to a block in autophagy. Depletion of VMP1 led to the presence of altered mitochondria in both HeLa and Cos-7 cells although the phenotype was more pronounced in the later (Fig 8A). Some mitochondria appeared longer and/or wider than with the control siRNA. The level of the mitochondrial marker Tim23, a protein from the inner mitochondrial membrane, was not grossly affected as determined by densitometry of three independent experiments, indicating that total amount of mitochondria is not largely affected (Fig 8A). This phenotype was clearly different from the one observed in Atg5-depleted cells, in which mitochondria appeared to be fragmented. Then, TEM was used to get a more accurate insight into the mitochondrial defects of VMP1-depleted Cos-7 cells. Alterations in mitochondrial size and morphology confirmed the confocal results. Some mitochondria showed altered size and most had dilated cristae or even lack of cristae (Fig 8B). We then analyzed possible defects in ER-mitochondrial contacts and remarkably the number and the length of ER-mitochondrial contact sites were increased in VMP1-depleted cells (Fig 8B). Morphometric quantifications of 250 mitochondria features from two independent inhibitions showed that the average number of contacts doubled in the VMP1-depleted cells (0.68 versus 1.38) and the proportion of mitochondrial membrane in contact with ER increased from 5.9% in control cells to 19% in depleted cells (Fig 8C). These results demonstrate that VMP1 is not functioning as a tether in these contacts but rather as a regulator of the extension and frequency of ER-mitochondria MCS.

## Discussion

Our results show that VMP1 is a surprisingly ubiquitous protein associated with a wide range of events in mammalian cells as it forms micro-domains associated with ER remodeling, ER-organelle contacts and organelle fissions. We have also shown that VMP1 functions as a negative regulator of the ER-mitochondria contact size. The accumulation of autophagosome markers on the ER and the abnormal distribution of lipid droplets in VMP1-depleted cells suggest that VMP1 could be required to restrict MCS allowing the correct size and/or dissociation of MCS in a variety of ER-organelle interactions. The altered morphology of the ER tubules in VMP1-depleted cells also strongly suggests that VMP1 play a role in shaping the ER structure.

A truncated form lacking a putative conserved amphipathic helix at the N-terminal cytosolic region (ΔNt-VMP1-GFP) does not form ER puncta. The presence of amphipathic helices alter the curvature of the membrane due to the partial insertion into the lipidic bilayer (e.g. Epsin, Arf, Sar1, alpha-synuclein) [37]. It is tempting to speculate that VMP1 could restrict the contacts by regulating the ER curvature. Other mechanisms of membrane curvature could be involved, such as the insertion of loops, as demonstrated for Caveolin-1 and reticulons, or the presence of conical lipids such as phosphatidylinositol phosphates (PtdIns) [37]. Interestingly, the absence of Vmp1 in Dictyostelium leads to the aberrant accumulation of PtdIns3P at the ER that could potentially alter its curvature [25]. Further biophysical studies on VMP1 structure and membrane curvature will be necessary to address this hypothesis and the role of the N-terminal region in the function of VMP1.

The pleiotropic defects associated with VMP1 depletion in model organisms suggested a more general function for this protein [38]. Our studies may now explain the apparently disparate phenotypes observed in different organelles such as mitochondria and chloroplasts in *Chlamydomonas* [26]; ER, Golgi and contractile vacuole in *Dictyostelium* [18], and processes as diverse as cytokinesis, secretion and endocytosis [18, 20, 26, 39, 40]. All these organelles and processes can potentially be affected by altered MCS as these contacts regulate the traffic of lipids, Ca2+ and signaling molecules [1]. Consequently, the impact of VMP1 depletion can be quite complex and cell-type specific as it may alter simultaneously the homeostasis of multiple processes. The specific defects associated with the lack of VMP1 in a given organelle might be the result of altered MCS size that could affect organelles in different ways [1]. The LD phenotype associated with the lack of VMP1 is intriguing since it suggests a role of VMP1 in LD size and distribution. A similar effect on LD has been reported in Atg2-depleted cells but the mechanism of this regulation remain elusive [27].

An additional novel aspect of the present study is the clarification of the conflicting results in the literature regarding the effect of VMP1 depletion on autophagy. Molejon *et*. *al*. reported that VMP1-depleted HeLa cells have an early block in autophagy due to the lack of recruitment and activation of the Class III PtdIns3P kinase [41]. However, the results of Mizuhima´s group in HeLa cells [21] and our previous results in *Dictyostelium* [25] showed that the lack of VMP1 does not prevent the recruitment of the autophagic machinery and the generation of PtdIns3P, but rather the opposite, since it results in an abnormal accumulation of PtdIns3P and aberrant omegasome morphology. Our results here, using HeLa and Cos-7 cells support and confirm the second model. We conclude that the lack of VMP1 leads to a distinct phenotype not shared by other Atg proteins [21]. Although in terms of localization VMP1 is the most upstream element to which the autophagic machinery is recruited [34], its absence leads to a late defect consisting in the persistent and aberrant accumulation of the autophagic machinery and the presence of large omegasomes [21, 23, 25]. This is supported by the persistent colocalization of the ER with autophagic structures as shown in Fig 6.

Autophagy requires complex membrane arrangements such the formation of the ER-derived omegasomes and also the presence of MCS with different organelles. Therefore, the unusual defects in autophagy observed in VMP1-depleted cells now become clearer. Our model proposes that VMP1 puncta is present in close proximity to different organelles prior to the recruitment of autophagic proteins, which explains the presence of puncta irrespective of growth and starvation conditions [21]. Only a small proportion of VMP1 puncta are engaged in autophagy during starvation conditions and these correspond mainly to VMP1/ER-Mitochondria contacts. Interestingly the autophagic machinery will be recruited to these ER sites in a VMP1-independent manner. Indeed our results indicate that a proportion of the accumulated LC3-labeled structures present in VMP1-depleted HeLa cells are in closed proximity with ER and mitochondria suggesting that they are trapped in abnormal autophagosome assembly sites (Fig 6C). Subsequently, VMP1, perhaps by restricting the size of the ER-mitochondria MCS (and may be other ER-organelle contacts) could modulate the correct spatial and temporal generation of PtdIns3P signaling, the omegasome morphogenesis and the recruitment of the different membrane sources (Fig 8D). This model is consistent with the observed phenotypes of VMP1-depleted cells described above. Interestingly, the lack of mitofusin-2, a protein involved in ER-mitochondria contact sites, causes an increase in the size of the contacts [42] and impairs starvation-induced autophagy [43]. Why these VMP1-enriched ER domains are preferable regions for autophagosome formation is not known and will justify further investigations. We can not rule out a possible more direct role of VMP1 in autophagy by modulating the ER structure for correct omegasome formation since our results in Cos-7 and in *Dictyostelium* [18] suggest an additional role in ER morphology.

In conclusion, our results radically change the view of VMP1 function, highlighting the importance of MCS in autophagy and organelle's function and propose that VMP1 is present in close proximity to organelles and autophagosome assembly sites, and regulates the size of MCS by a still unknown molecular mechanism.

## Supporting Information

S1 Fig VMP1 depletion leads to accumulation of autophagic structures at the ER. (A) Analysis by western-blot of the levels of VMP1 and Atg5 protein after transfection of the corresponding siRNAs. (B) LC3 turnover assays of control and VMP1-depleted HeLa and Cos-7 cells. VMP1 depletion block autophagy in both cell types although the effect is more pronounced in HeLa cells.
(EPS)

S1 Movie Sliding events at the ER-VMP1 puncta 1. (MP4)

S2 Movie Sliding events at the ER-VMP1 puncta 2. (MP4)

S3 Movie Sliding events at the ER-VMP1 puncta 3. (MP4)

S4 Movie Tethering VMP1 mitochondria. (MP4)

S5 Movie Tethering VMP1 lipid droplets. (MP4)

S6 Movie Tethering VMP1 early endosomes. (MP4)

S7 Movie Tethering VMP1 late endosomes. (MP4)

S8 Movie Fission Mitochondria 1. (MP4)

S9 Movie Fission Mitochondria 2. (MP4)

S10 Movie Fission late endosomes. (MP4)

S11 Movie VMP1 and lipid droplets. (MP4)
